# Phylogenetic Heatmaps Highlight Composition Biases in Sequenced Reads

**DOI:** 10.3390/microorganisms5010004

**Published:** 2017-01-24

**Authors:** Sulbha Choudhari, Andrey Grigoriev

**Affiliations:** Department of Biology, Center for Computational and Integrative Biology, Rutgers University, Camden, NJ 08102, USA; sulbha@scarletmail.rutgers.edu

**Keywords:** nucleotide composition, metagenomics, sequencing bias, computational analysis, genome sequencing

## Abstract

Due to advancements in sequencing technology, sequence data production is no longer a constraint in the field of microbiology and has made it possible to study uncultured microbes or whole environments using metagenomics. However, these new technologies introduce different biases in metagenomic sequencing, affecting the nucleotide distribution of resulting sequence reads. Here, we illustrate such biases using two methods. One is based on phylogenetic heatmaps (PGHMs), a novel approach for compact visualization of sequence composition differences between two groups of sequences containing the same phylogenetic groups. This method is well suited for finding noise and biases when comparing metagenomics samples. We apply PGHMs to detect noise and bias in the data produced with different DNA extraction protocols, different sequencing platforms and different experimental frameworks. In parallel, we use principal component analysis displaying different clustering of sequences from each sample to support our findings and illustrate the utility of PGHMs. We considered contributions of the read length and GC-content variation and observed that in most cases biases were generally due to the GC-content of the reads.

## 1. Introduction

In recent years, metagenomics has emerged as a powerful tool involving the study of the genome of microbial communities by sequencing microbial DNA extracted directly from environmental samples. Advancements of next generation sequencing technologies (NGS) have provided researchers with the ability to study entire microbial communities from the diverse environment and discover new organisms. One of the primary applications of metagenomics is 16S rRNA surveys to study the phylogeny and taxonomy of samples from complex microbial communities. Sequencing technologies utilize the hypervariable regions of 16S rRNA for the identification of different bacterial species [1].

Although metagenomics and high-throughput sequencing have expanded our knowledge in the field of microbiology by directly accessing the microbial community genomes, there are still uncertainties in the data. There are many challenges in analyzing metagenomic data, including the assessment of microbial abundance in environmental samples using frequency of occurrence of an organism’s DNA observed in sequencing reads [2]. Such frequencies and corresponding abundance estimates depend significantly on the DNA extraction and sequencing protocols used. Earlier studies have shown that the sequencing technologies have different biases, depending on the approach adopted to obtain sequence data. Several groups have investigated the bias imposed by (i) various DNA extraction protocols using environmental samples [2,3] and by (ii) NGS platforms based on phylogenetic profiling of sequencing reads [4,5]. A recent study revealed different DNA extraction and PCR amplification protocols impose more bias than sequencing and taxonomic classification in 16S analysis [6]. The differences in DNA sequencing protocol may introduce biases in the resulting sequences, as highlighted by a comparison of Illumina and Roche 454 platforms in an analysis of a freshwater planktonic community [7]. The study revealed the bias towards A’s and T’s over C’s and G’s in homopolymer sequences generated by Roche 454. With Illumina, these patterns were less common, and the errors were more randomly distributed. The sequences produced by different platforms introduce systematic biases and unique patterns of biased sequence coverage [8]. Furthermore, the approaches used for the taxonomic classification of metagenomic reads also introduced their own limitations [9]. However, a sequence composition-based comparison between different sequencing platforms has not been done yet.

Recently, we performed a sequence-based comparison of two geographically distinct glacier metagenomes [10], that of an Alaskan glacier [11] and an Alpine glacier [12], and observed a striking differences between them. Not only have we seen a significant difference in the numbers of operational taxonomic units (OTU) between the two samples, but a sequence composition of their reads was very dissimilar [10]. Although the same type of starting material (ice/snow) was used for the two samples, different sequencing approaches were used to obtain data. This motivated us to develop a method for compact consistent visualization of the difference between the samples based on the composition of the produced sequence reads.

Here we describe such a method (which we named “phylogenetic heatmaps”, or PGHMs) for detecting sequence-based bias in metagenome sequence data generated by different sequencing platforms. In general, PGHMs can be used for a comparison of nucleotide composition of two sets containing sequences that can be classified into the same classes, e.g., phylogenetic groups, and is suited for metagenomics samples. For evaluation we examined the sequences from mock communities generated by one platform, but using different protocols for DNA extraction [3]. Additionally, we compared two very diverse and rich metagenomes (human gut and soil) generated by the same sequencing platform. Furthermore, we analyzed another human gut metagenome, which was a part of the human microbiome project generated by two different platforms. Finally, we analyzed three different soil metagenomes [13,14] generated by different platforms to study the sequence composition in an environmental sample.

The general question is how to use the information obtained via different technologies for the comparative purposes. We took the sequences generated by different approaches and classified them by binning into distinct taxonomic groups. We calculated nucleotide word frequencies in these sequencing reads and displayed the results using PGHMs and principal component analysis (PCA). We observed striking differences in the nucleotide composition of the reads generated by different sequencing platforms and related it to the PCA load factors and GC composition of the hypervariable regions of 16S rRNA. We also examined the load factors of the principal component that gave the maximum variance to the data to identify which particular word nucleotide was responsible for the difference. Additionally, the GC-content of the data informed us about the sample GC-bias. We observed that sequence composition of reads in a metagenome varied based on different steps in the selected sequencing methodology, and we also noticed differences in the sequences generated for the same variable region from a single species.

## 2. Material and Methods

### 2.1. Data Sets

All data used in current study was downloaded from Sequence Read Archive (SRA, https://www.ncbi.nlm.nih.gov/sra) that was generated in previously published studies. The data for mock communities was taken from SRA that was generated by the same sequencing platform, however, with different DNA-extraction protocols used for samples preparation. Out of four protocols analyzed in the study, only two (named Q and BB in the original publication [3]) were considered here for the analysis. The DNA has been extracted from mock communities of seven representative oral bacteria and sequenced via 454 (SRP039007). Next, we analyzed two very diverse metagenomes, human gut and soil metagenomes [13], sequenced by the Illumina MiSeq to study the sequence diversity of two metagenomes sequenced by the same platform. Stool samples for gut metagenome have been studied previously in the context of the bacterial geography of human gastrointestinal tract [15]. A temperate deciduous forest (TDF) soil sample was selected from the Canadian MetaMicrobiome Library [16]. In another study, both the gut and soil metagenome data have been used for evaluating the bias of Illumina-based bacterial 16S rRNA gene profiles [16]. The V3 region of 16S rRNA of the human gut (ERR567417) and soil metagenome (ERR567426) was downloaded from SRA. In our study, the human gut data will be referred as “human gut I” and soil data will be identified as “soil I”.

The other human gut data used in the analysis was taken from different studies that investigated the human gut microbiome using 454 GS FLX Titanium (Roche Diagnostics, Basel, Switzerland) and Illumina MiSeq sequencing technologies (Illumina, San Diego, CA, USA). The hypervariable regions of 16S rRNA gene were sequenced using DNA extracted from various human gut samples. For each platform, sets of eight sequence data sets, SRR1029510-17 (454) and SRR1029468 -75 (Illumina) were downloaded from SRA database (Bioproject: PRJNA46315). This human gut will be called as “human gut II” in this study. The different soil metagenomes data for the environmental sample used for analysis have been generated from 454 GS FLX, Ion Torrent PGM (Thermo Fisher Scientific, Waltham, MA, USA), and Illumina Genome Analyzer IIx platforms (Illumina, San Diego, CA, USA). The accession numbers of the soil data downloaded from SRA were: SRX404651 (454), SRX481936 (Ion Torrent), and ERX093708 (Illumina) and in present study these soil metagenomes will be identified as “soil II”. These data sets contained sequences from various hypervariable regions of 16S rRNA and sequence composition of each region is different, which minimizes the possibility of direct comparisons. To overcome the limitation, we used the same hypervariable region for the comparisons in each dataset to minimize any additional bias in the analysis.

### 2.2. Data Processing

All reads from the different platforms were quality filtered, and the sequences that passed the Phred scores greater than 20 and did not contain any “N”s were retained. A Python script was written to extract only the unique hypervariable regions of the 16S rRNA gene from filtered reads, excluding the barcodes and primer sequences in order to compare different platforms based on unique hypervariable region. This resulted in slightly different read lengths in all the samples except ERX093708, where the first 100 bp of amplified V5–V6 hypervariable region that represented V5 region was used for the analysis. Table 1 represents a summary of the data screening details of each platform/sample used in our study.

### 2.3. Principal Component Analysis (PCA)

Oligonucleotide word frequencies are frequently used in metagenomic applications (e.g., phylogenetic tree construction [17], metagenome binning [18]). Here, we adopted a similar technique to analyze the sequence composition of hypervariable regions of 16S rRNA fragments using word frequencies. We grouped different hypervariable regions of the reads sequenced from different sequencing platforms based on, di-, tri- and tetra- nucleotide patterns. Subsequently, PCA was applied on the nucleotide frequencies by reducing the feature dimensionality to two while still retaining the most informative features. Each DNA fragment or read is represented by a feature vector (*K* = 336), where each factor in a vector encodes the frequency of a particular, di-, tri- or tetramer occurring in a read. The data are then transformed into principal components, which are ordered according to their corresponding variances. To observe similarities and differences, the projections on the two principal components are plotted, highlighting clustering of the sequences. The coefficients of variables (i.e., load factors), which in this case are the 336 word frequencies of the first principal components, provided information on how nucleotide words contributed to the variance in the data. A histogram was generated with top 20 and bottom 20 load factors and their corresponding frequencies, and the GC-content was also calculated for each dataset.

The taxonomic classification of extracted regions of 16S rRNA was performed by mothur [19] using default settings and the taxonomy outline from SILVA [20] database. We then used the k-nearest neighbor consensus and BLAST [21] for taxonomic classification of the reads.

### 2.4. Phylogenetic Heatmaps

Since PCA has no stochasticity constraints, the effectiveness of the clustering of different phylogenetic taxa in PCA was estimated by calculating the Pearson’s correlation coefficient (*r*) of all the sequences with each other and taking the average correlation coefficient between the different groups within a sample and across samples. Correlation coefficients are not additive, therefore we transformed *r* values to Fisher *z* = ln((1 + *r*)/(1 − *r*))/2 to produce additive quantities. Once *r* values were converted into *z*-scores and an arithmetic mean score *z* was computed, Fisher mean value was calculated as *R* = (e^*z*^ − e^−*z*^)/(e^*z*^ + e^−*z*^).

The mean r for all group pairs is represented in a phylogenetic heatmap (PGHM). A concentric heatmap is generated, where the inner circle displays the correlation between the same phylogenetic groups in two samples, and the outer circle shows how correlated are the closest but different phylogenetic groups in the same sample. The procedure for generating a heatmap is as follows.

Let *N* be the number of different phylogenetic groups analyzed (normally the most numerous taxa in the sample, we use *N* = 4 here but any number *N* > 1 can be used). First, the inner circle is generated, that consists of *N* wedges representing (as shades of gray) mean correlation between the same groups from two different samples. Then, for each group in an inner wedge the nearest group in its own sample is found, thus generating two outer wedges, shaded according to mean correlation coefficients between these groups from the same samples. As illustrated below, PGHMs clearly indicate compositional biases in a compact visual form.

To access the statistical significance of the correlation we also computed z-scores comparing the distribution of correlation coefficients between all sequences from the pair of bacterial groups from an inner wedge and the same distribution from the pair of bacterial groups in each of the two corresponding outer wedges. In all cases analyzed below the difference between mean correlation coefficients was significant (*p* < 0.001), except for one case mentioned specifically.

### 2.5. Availability

The software implementing our approach for metagenomics in Python, together with examples and user instructions is freely available at grigoriev.rutgers.edu/software/PGHM-meta/ and as a Supplementary File. The PGHM was developed on linux Fedora (version 22, Red Hat, Raleigh, NC, USA). The current Python implementation is fast, the computation time for the example dataset, provided with the software is 3.5 min on a system with a 2.50 GHz Intel i5-3210M CPU processor and 8 GB RAM. 

## 3. Results and Discussion

### 3.1. Positive and Negative Controls

**Figure 1:**
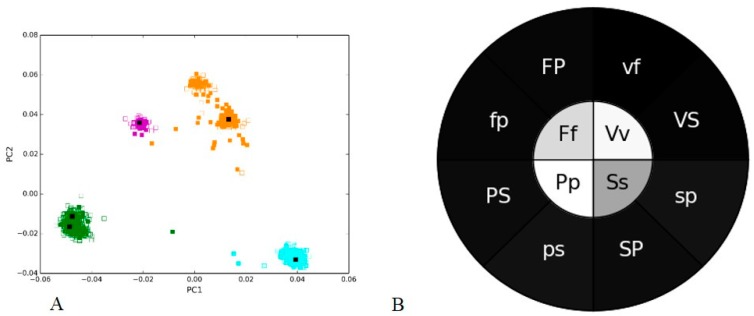
V1–V2 hypervariable regions of 16S rRNA of bacterial species using different DNA-extraction protocols. (**A**) Nucleotide word frequency PCA (Principal Component Analysis) plot of sequence data from Q (solid boxes) and BB (empty boxes) protocols, with phylogenetic classification shown by color (*Fusobacterium* = green, *Porphyromonas* = magenta, *Streptococcus* = orange, *Veillonella* = turquoise); (**B**) Phylogenetic heatmap: the heatmap inner circle shows the mean Pearson’s correlation computed between the same bacterial group from Q and BB protocols, and the wedges in outer circle represent the groups with the highest correlation within one protocol (uppercase letters indicating sample from protocol Q and lowercase—from protocol BB, for representation the first letter of each name is used). In the inner circle the lowest correlation coefficient is 0.71 for Ss, since two strains are considered together. In the outer circle, all coefficients are ≤0.11, indicating no correlation.

We first evaluated the extent of composition bias generated in sequencing experiment on single known sequences taking data from a recent study that analyzed bacterial species representation in a mock community while using different DNA extraction methods [3]. The mock community included *Streptococcus oralis* 34, *Streptococcus mutans* NCTC 10449, *Fusobacterium* ATCC 10953, *Porphyromonas gingivalis*, and *Veillonella parvula* PK 1910, with the V1–V2 hypervariable region of the 16S rRNA used for analyzing the sequence composition and for phylogenetic classification. As in the sections below, we considered broader phylogeny and analyzed both *Streptococcus* species together. This data was used as an “ultimate positive control”, and we observed that each species, predictably, formed a distinct cluster in the PCA plot based on the nucleotide word frequencies. For both DNA extraction protocols (solid or empty boxes), sequences form the same species clustered together (Figure 1A). We call this effect “phylogenetic clustering” throughout the paper, in contrast to cases of “sample clustering” (considered in sections below), where phylogenetically distant groups from the sample clustered together.

The clusters of each species scattered distinctly (Figure 1A) along the PC1 axis, which explained about 45% of variability, and PC2 (31% of variability), but the same species clusters were overlapping for each protocol. PC1 highlighted a separation between different species based on the nucleotide composition of the resulting sequences, and PC2 provided a separation between *Porphyromonas* and *Streptococcus* with the other two species, *Fusobacterium* and *Veillonella.* The two species of *Streptococcus* formed two separate clusters, depicting the difference in the sequence composition of two species. Each cluster represented a specific V1–V2 region from a single species, and ideally, one should expect a single point for each species rather than a cluster of sequences; instead we observed variance in the data. Notably, the most abundant sequences of species from each protocol are in the center of each cluster (black solid boxes), and the observed scatter represents likely sequencing errors and not the effects of length variation since length distributions are very narrow (Figure S1).

We then investigated the effectiveness of clustering in PCA analysis using our PGHM methodology for the two samples. We calculated the mean Pearson’s correlation coefficient for all possible pairs of sequences within each cluster and between the clusters, as detailed in the Methods. These means were then converted into different shades of gray, with darker shade corresponding to a lower (between 0 and 0.11 in this case) and lighter shade to a higher correlation coefficient (between 0.71 and 1), respectively, and plotted them in a concentric heatmap (Figure 1B).

The inner circle of the heatmap shows the correlation between the same bacterial species in two samples, marked by the first letter of the name in upper and lower case (e.g., “Pp” in case of *Porphyromonas*). The wedges in the outer circle of the heatmap represented the correlation between the bacterial species that were found closest to each other in a PCA plot within a sample. E.g., a “PS” wedge in uppercase would correspond to the mean correlation for all pairs of sequences between *Porphyromonas* (P) an*d Streptococcu*s (S) for one DNA-extraction protocol (namely, Q), and, “ps” in lowercase for another protocol (BB). In case of no bias, the same species from different samples are expected to be identical (or closer to each other, given the sequencing noise) in nucleotide composition to each other than to other species. This phylogenetic clustering is seen in the PCA plot (Figure 1A), and is clearly illustrated by the lighter shade of the inner circle (i.e., higher within-species correlation) compared to the outer circle (Figure 1B), despite the isolated cases of *Streptococcus* reads being closer to *Porphyromonas* reads. If such cases represent incorrect assignment, the PGHM method is not affected by them, as this example shows. This method is not limited to comparisons between such identical communities, in fact it could be used to compare any two samples that share common bacterial groups.

**Figure 2:**
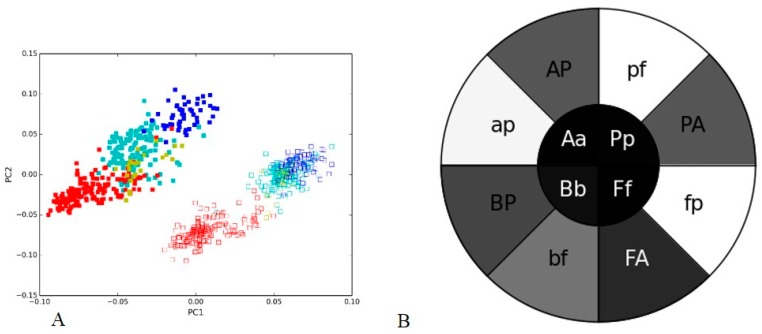
The V3 and V4 hypervariable regions of 16S rRNA of bacterial groups from soil metagenome I generated through Illumina MiSeq: (**A**) Nucleotide word frequency PCA of sequence data of V3 (solid boxes) and V4 (empty boxes) of 16S from soil I metagenome with phylogenetic classification at the phylum-level (*Actinobacteria* = blue, *Bacteroidetes* = red, *Firmicutes* = yellow, *Proteobacteria* = turquoise); (**B**) Phylogenetic heatmap: the heatmap inner circle shows the mean Pearson’s correlation computed between V3 and V4 region from the same bacterial group, and the wedges in outer circle represent the groups with the highest correlation within one region (uppercase letter indicating a group from V3 region and lowercase letter—a group from V4).

The method was further tested for an opposite case (negative control) of two different hypervariable regions from soil metagenome I sequenced with Illumina MiSeq. The most dominant bacterial groups, *Bacteroidetes* and *Firmicutes* (which constitute the vast majority of the dominant human gut microbiota [22]), as well as *Actinobacteria* and *Proteobacteria* that are abundant in environmental samples [23] were selected for the analysis. The V3 and V4 hypervariable regions of 16S were analyzed for the sequence composition and taxonomic classification. As expected both the variable regions formed separate clusters in PCA (Figure 2A) with subclustering of different bacterial groups in each cluster. The inner circle displayed in the PGHM (Figure 2B) is darker compared to the outer one (exact opposite to that found in the positive control).

### 3.2. Human Gut I and Soil I Metagenome

**Figure 3:**
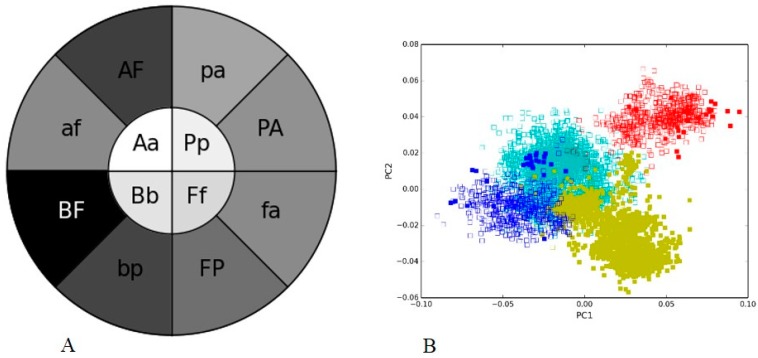
V3 hypervariable regions of 16S rRNA of bacterial groups from two diverse metagenomes generated through Illumina MiSeq: (**A**) Phylogenetic heatmap: the heatmap inner circle shows the mean Pearson’s correlation computed between the same bacterial group from human gut and soil metagenomes, and the wedges in outer circle represent the groups with the highest correlation within one metagenome (uppercase letter indicating group name from human gut and lowercase letter from soil metagenome); (**B**) Nucleotide word frequency PCA of sequence data from human gut (solid boxes) and soil (empty boxes) metagenome with phylogenetic classification at the phylum-level (*Actinobacteria* = blue, *Bacteroidetes* = red, *Firmicutes* = yellow, *Proteobacteria* = turquoise).

We compared two diverse metagenomes generated by the same platform within the same experimental framework aimed to understand Illumina sequencing bias in a recent study [16]. Here, we analyzed the human gut I and soil I metagenome sequenced by Illumina MiSeq and the same groups were selected as used in the negative control. The V3 hypervariable regions have been sequenced in both data sets and were utilized for analyzing the sequence composition and taxonomic classification. We observed a lighter shade of the inner circle compared to the outer circle in the PGHM (Figure 3A), with each bacterial group represented by its first letter of the name in upper and lower case (e.g., ***A*** or ***a*** in case of *Actinobacteria*). This indicated that the same phylogenetic groups in two samples are closer to each other than to other groups based on the nucleotide composition within a sample. The phylogenetic clustering observed in the PCA plot (Figure 3B) is consistent with the heatmap “doughnut” (Figure 3A), as the clusters of sequences from two metagenomes overlap by phylogeny (Figure 3B). Hence, our visualization approach can capture the sequence composition trends not only in simple mock communities but also in complex metagenomes.

### 3.3. Human Gut II: Illumina vs. 454

**Figure 4:**
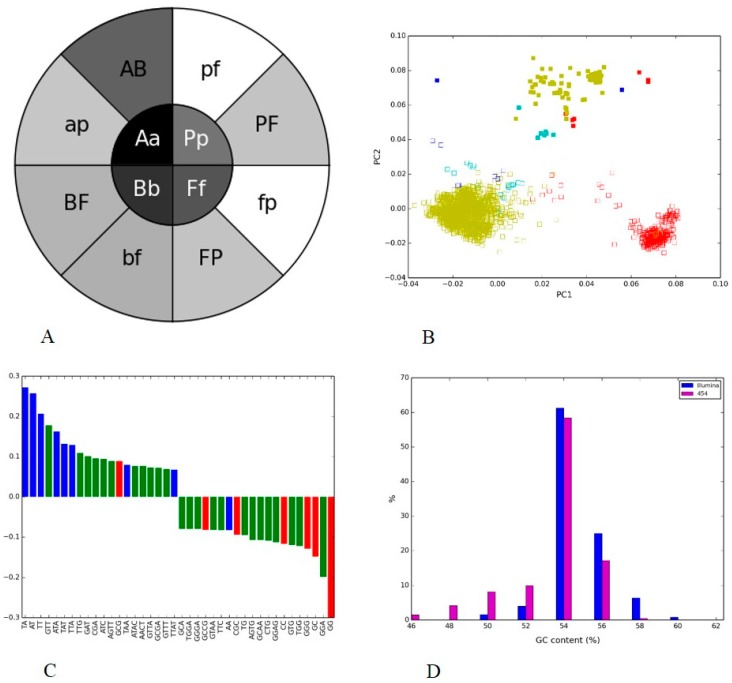
V4 hypervariable regions of 16S rRNA of bacterial groups from two human gut metagenomes generated through Illumina MiSeq and 454: (**A**) Phylogenetic heatmap: the heatmap inner circle shows the mean Pearson’s correlation computed between the same bacterial group from both the platforms, and the wedges in outer circle represent the groups with the highest correlation within one platform (uppercase letter indicated phylum from Illumina and lowercase letter represented phylum from 454); (**B**) Nucleotide word frequency PCA of sequence data from human gut generated via Illumina MiSeq (solid boxes) and 454 (empty boxes) with phylogenetic classification at the phylum-level (*Actinobacteria* = blue, *Bacteroidetes* = red, *Firmicutes* = yellow, *Proteobacteria* = turquoise); (**C**) Top 20 and bottom 20 load factors of the first principal component and their corresponding frequencies. Different colors indicate GC content of nucleotide words (red = 100% GC, green = less than 100% and more than 0% GC, blue 0% GC); (**D**) GC content distribution for reads produced by each platform.

We then compared human gut metagenome data sets sequenced by 454 and Illumina technologies. The variable V4 region of the 16S rRNA was common between the samples, and this region was extracted from the reads of sequenced microbiomes for identification of phylogenetic groups and for analyzing nucleotide composition patterns using PGHM and PCA. The PGHM showed a darker shade of gray in the inner circle compared to the outer circle (Figure 4A), indicating that the sequence composition of a phylogenetic group was closer to other groups within its sample than to the same group in the second sample. The difference between the distribution of correlation coefficients between all sequences from the pair of bacterial groups from an inner wedge and the same distribution from the pair of bacterial groups in each of the two corresponding outer wedges was statistically significant in all cases but one. The difference between mean correlation coefficients between the pair of *Actinobacteria* in the inner wedge and the pair *Actinobacteria* and *Bacteroidetes* from Illumina was not significant (*p* = 0.29). The “sample clustering” was consistent with the PCA plot where phylogenetically distant groups from the sample lumped together. The scatter plot of PC1 vs. PC2, which together accounted for 43% of the variability, showed a clear separation between the two platforms along the PC2 axis (Figure 4B).

The different bacterial groups were scattered along the PC1 axis and grouped according to their sequencing platform along the PC2 axis. We also observed that the clusters from Illumina occupied the positive PC1 axis whereas the 454 clusters scattered along this axis. Although different phyla formed separate taxonomic groups in the reduced sequence composition space, they remained in the vicinity of their sequencing platform counterparts (Figure 4B).

To investigate the reason for this difference, we compared the load factors of the first principal components to see if specific nucleotide words could explain the observed variance. Principal component load factors indicate the contribution of each variable (in this case, nucleotide word frequency) in accounting for variability in the principal component. We plotted the top 20 positive and top 20 negative load factors as histograms for PC1 (Figure 4C), coloring the bars according to GC content of the nucleotide words (e.g., the color red indicates all-GC words, with 100% G or C; the color blue indicates non-GC words, with 0% of G or C; and the color green -indicates all intermediate values).

We observed that the sample/platform separation was very clearly provided by PC2 and some species separation—by PC1. One word, “GG”, provided the largest contribution to the PC1, and its frequency distribution pattern played a role in differentiating phylogenies on both platforms. Figure 4B showed clusters of 454 (except *Bacteroidetes*) were <0 on the PC1 axis, and negative axis in Figure 4C was dominated by GC-rich (red bars) nucleotide words. This indicated that the two technologies clearly differ in their ability to capture sequences with different GC content, with “GG” being the main contributor to this difference.

The GC-content distributions of the sample sequences (Figure 4D) showed Illumina and 454 data sets follow the same distribution pattern with a peak at 54.5% GC. PCA load factors (Figure 4C) provided a better insight into the contribution of specific GC-rich/poor nucleotide words while GC-content plots showed more general trends. A striking indicator of bias, for example, was the sequence composition of bacterial group *Actinobacteria* produced by two different platforms. These two *Actinobacteria* clusters were further apart than sequences of *Actinobacteria* and other bacterial groups (*Bacteroidetes*, *Firmicutes*, and *Proteobacteria*) generated by the same platform (Figure 4B). Contrary to the expectation that the sequences of the same bacterial group would be similar in composition regardless of sequencing platform utilized for generating data we found that the sequences of unrelated bacterial groups were compositionally similar to each other if they were generated by the same sequencing technology.

### 3.4. Soil Metagenome II: Illumina, Ion Torrent and 454

**Figure 5:**
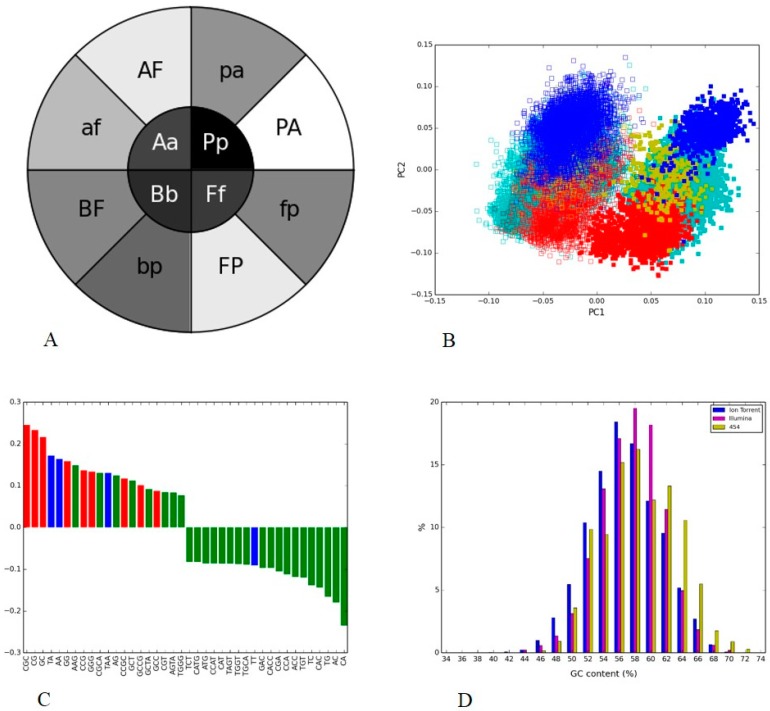
V5 hypervariable regions of 16S rRNA of bacterial groups from two soil metagenomes generated through Ion Torrent and Illumina Genome Analyzer: (**A**) Phylogenetic heatmap: the heatmap inner circle shows the mean Pearson’s correlation computed between the same bacterial group from both the platforms, and the wedges in outer circle represent the groups with the highest correlation within one platform (uppercase letter indicated phylum from Ion Torrent and lowercase letter represented phylum from Illumina); (**B**) Nucleotide word frequency PCA of sequence data from soil metagenome generated via Ion Torrent (solid boxes) and Illumina Genome Analyzer (empty boxes) platforms with phylogenetic classification at the phylum-level (*Actinobacteria* = blue, *Bacteroidetes* = red, *Firmicutes* = yellow, *Proteobacteria* = turquoise); (**C**) Histograms of the first component; (**D**) GC-content.

**Figure 6:**
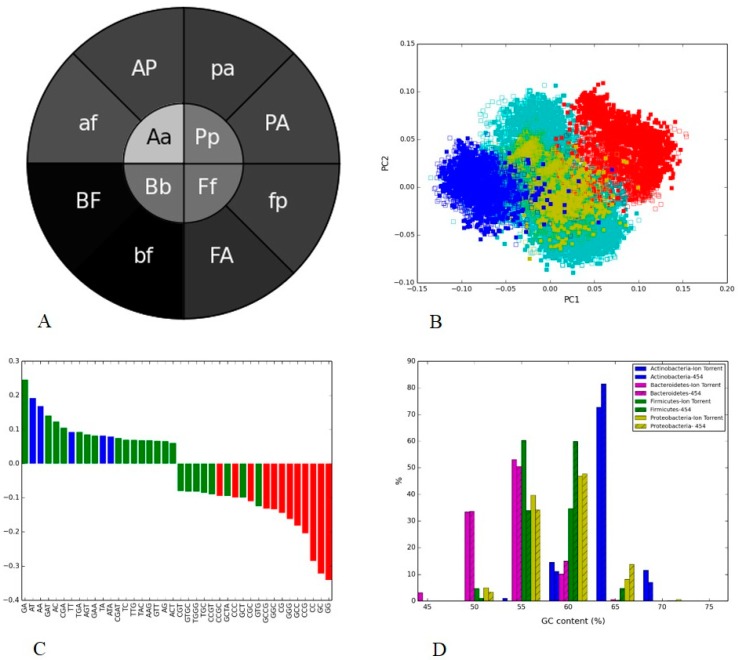
V5 hypervariable regions of 16S rRNA of bacterial groups from two soil metagenomes generated through Ion Torrent and 454. (**A**) Phylogenetic heatmap were displayed via heatmap, where the inner circle showed the mean of Pearson’s correlation (Fisher transformation) computed between the same bacterial group from both the platforms, and the wedges in outer circle represent the correlation within one platform (the uppercase letter indicated phylum from Ion Torrent and lowercase letter represented phylum from 454); (**B**) Nucleotide word frequency PCA of sequence data from soil metagenome generated via Ion Torrent (solid boxes) and 454 (empty boxes) platforms. PCA orientation with phylogenetic classification at the phylum-level (*Actinobacteria* = blue, *Bacteroidetes* = red, *Firmicutes* = yellow, *Proteobacteria* = turquoise); (**C**) Histograms of the first component; (**D**) GC-content.

We further considered platform-specific biases in environmental metagenomes using soil samples sequenced by 454, Illumina, and Ion Torrent platforms. We performed two pairwise comparisons: Illumina vs Ion Torrent (Figure 5), and Ion Torrent vs 454 (Figure 6). The common V5 hypervariable region of 16S rRNA was used for the calculation of nucleotide frequencies, and phylogenetic classification. In the first case, we observed bias in the PGHM (Figure 5A) that was consistent with sample clustering in the PCA plot (Figure 5B), whereas phylogenetic clustering in PCA (Figure 6B) and “doughnut” heatmap were seen in the second case (Figure 6A).

The sample clustering observed in PCA plot in the first case was illustrated by PGHMs, where the inner circle was darker (i.e., lower within-taxa correlation) compared to the outer circle (Figure 5A). Unlike human gut, the sample separation from different platforms was very clearly represented by PC1 in PCA plot in the first case (Figure 5B). The GC-content of the sequences from the two samples was also much closer, with the peaks for each technology within 3% G + C, Figure 5D. By far, the top three load factors in the first component were all-GC nucleotide words (i.e., red bars; Figure 5C). This finding was in agreement with the arrangement of the cluster in the PCA plot, where the Ion Torrent solid boxes were in the positive half of the PC1 axis, indicating that this platform produced more GC-rich sequences compared to Illumina. Although this may seem contrary to the slightly higher mean GC content of Illumina vs. Ion (Figure 5D), a more detailed investigation of the top load factors revealed that the distributions of all high load factors in both samples were bi- or multi-modal (data not shown), with the top three all-GC nucleotide words favoring Ion Torrent compared to Illumina, and thus resulting in the separation of clusters along the PC1 axis (Figure 5B).

In contrast, phylogenetic clustering is seen in the PCA plot for sequences generated via Ion Torrent and 454, and is clearly highlighted by the PGHM where inner circle in the heatmap was lighter in shade compared to the outer one (i.e., high within-taxa correlation). This reflects a case of no sample bias, with slight (due to very close clustering in Figure 6A) but significant difference between the two circles. In this case, the sequences dispersed along PC1 axis, which accounted for around 20% of the total variance, and clustered into separate taxonomic groups in the reduced sequence composition space of PCA (Figure 6B).

When we considered the load factors of PC1 (as the separation was along this axis), we observed that the negative load factors were dominated by GC-rich nucleotide words (i.e., red bars). *Actinobacteria* occupied the negative domain on the PC1 axis, suggesting that this group was GC-rich. This finding was in agreement with the fact that *Actinobacteria* showed higher GC content on both platforms plot (Figure 6D). The GC% content distribution of 454 and Ion Torrent were closer, peaking at 60% GC and 55% GC, respectively (Figure 6D). However, the GC-content peak for the most distant clusters on PCA, *Actinobacteria*, and *Bacteroidetes* were 64% and 55%, respectively, in both platforms (Figure 6D). This finding was in agreement with these groups being GC-rich and GC-poor, respectively. *Proteobacteria* and *Firmicutes* overlapped, as they shared the similar GC–content in both platforms (58%–60%). We can conclude here that the main reason for different clustering on PCA plot was due to the difference in the GC-rich nucleotides in different phylogenies, as similar grouping for both platforms was observed. While read length distributions in the samples may potentially have an effect on the composition, it appears to be minimal in this (Figures S2–S4) and other cases (not shown) as such distributions are very narrow and thus affect only a few word frequencies. 

## 4. Conclusions

In summary, we conclude that the sequence composition of samples that were supposed to represent similar/comparable metagenomes varied depending on the sequencing technologies used and our method is efficient in detecting/finding such variation. The di-, tri- and tetramers well represented the sequence composition of the reads (and a very good correlation between binned sequences based on word frequencies and phylogeny was observed previously [24]). In the current study, we observed the expected PCA clustering by the sequence composition of the reads in the positive and negative controls, supported by the PHGMs. We also observed phylogenetic clustering and sample clustering in different cases, which appeared to be influenced by multiple experimental choices. We note that sample biases visualized with both PCA and PHGMs are not just platform-specific, but that other upstream processes, such as DNA extraction [2,3] and PCR amplification [25], can generate biases affecting the nucleotide composition of the resulting reads. Broad read length distributions in the samples may potentially have an effect on the composition, however, they appeared very narrow in all cases we considered in this study. Further, the choice of primers and library preparation method has been shown to generate sequences with substitutions, insertions and deletions towards certain motifs [26] and we observed the link between these biases, phylogenetic/sample clustering and GC content of the sample reads. Such biases can ultimately affect the precision of the downstream analysis tools and results of metagenomics studies [27]. Thus, one needs to exercise caution when comparing and combining metagenomic data produced by different technologies and utilize uniform methods for comparative studies. Our compact visual methodology of PGHMs may find further use in such analyses of multiple metagenome samples.

## Figures and Tables

**Table 1:** Data processing details of Next Generation Sequencing platforms used in this study.

Platform	Accession ^S^	Illumina			454			Ion Torrent		
		Raw Reads	Quality Filter	HV	Raw Reads	Quality Filter	HV	Raw Reads	Quality Filter	HV
Human Gut I	ERR567417	44,360 ^M^	19,677 ^M^	V3 ^M^						
Soil I	ERR567426	47,685 ^M^	14,604 ^M^	V3 ^M^						
Human Gut II	SRR1029468 ^I^	358,773 ^M^	124 ^M^	V4 ^M^	154,374	3215	V4			
	SRR1029510 ^454^									
Soil II	ERX093708 ^I^	42,864 ^GA^	26,385 ^GA^	V5 ^GA^	729,514	11,349	V5	514,848	11,052	V5
	SRX404651 ^454^									
	SRX481936 ^IT^									
Protocol Q	SRP039007				12,317	1050	V1–V2			
Protocol BB	SRP039007				13,724	3243	V1–V2			

^S^: Sequence Read Archive accession number; ^GA^: Illumina Genome Analyzer IIx; ^M^: Illumina MiSeq; ^I^: Illumina; ^IT^: Ion Torrent; HV: hypervariable regions of the SSU rRNA gene.

## Supplementary Materials

The following are available online at www.mdpi.com/2076-2607/5/1/4/s1, Figure S1: Length distribution for the samples in Figure 1 (colors as in Figure 1). Two peaks for *Streptococcus* correspond to the two species, Figure S2: Length distribution for the 454 sample in Figure 6 (colors as in Figure 6B), Figure S3: Length distribution for the Illumina sample in Figure 5 (colors as in Figure 5B), Figure S4: Length distribution for the Ion Torrent sample in Figure 5 and Figure 6 (colors as in Figure 5B and Figure 6B).
