# Supplementary material for: Phylogenetic Heatmaps Highlight Composition Biases in Sequenced Reads
Source: Microorganisms. 2017 Jan 24;5(1):4. doi: 10.3390/microorganisms5010004 (PMC5374381; doi:10.3390/microorganisms5010004)
Supplement: Supplementary file 1 [file microorganisms-05-00004-s001.pdf]

microorganisms

Supplementary Materials: Phylogenetic Heatmaps Highlight Composition Biases in Sequenced Reads

Sulbha Choudhari and Andrey Grigoriev

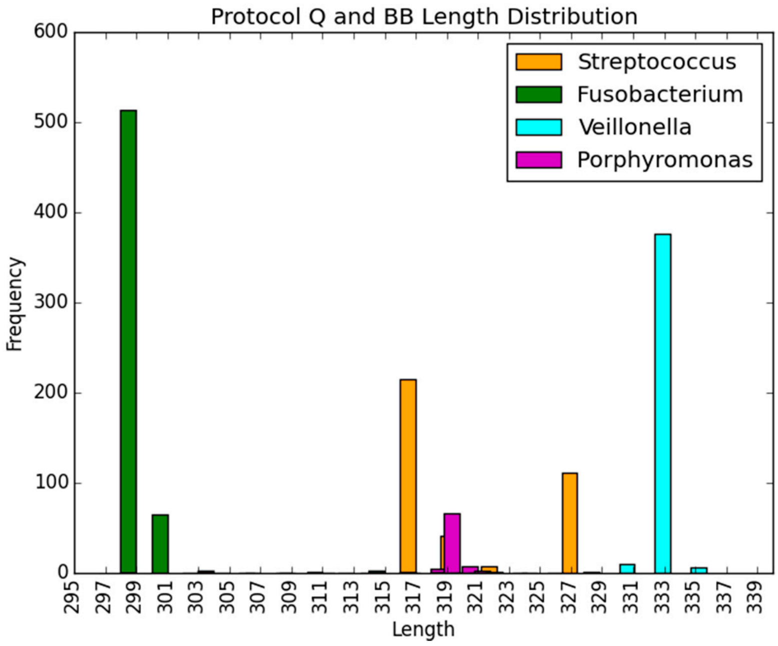

Figure S1. Length distribution for the samples in Figure 1 (colors as in Figure 1). Two peaks for *Streptococcus* correspond to the two species.

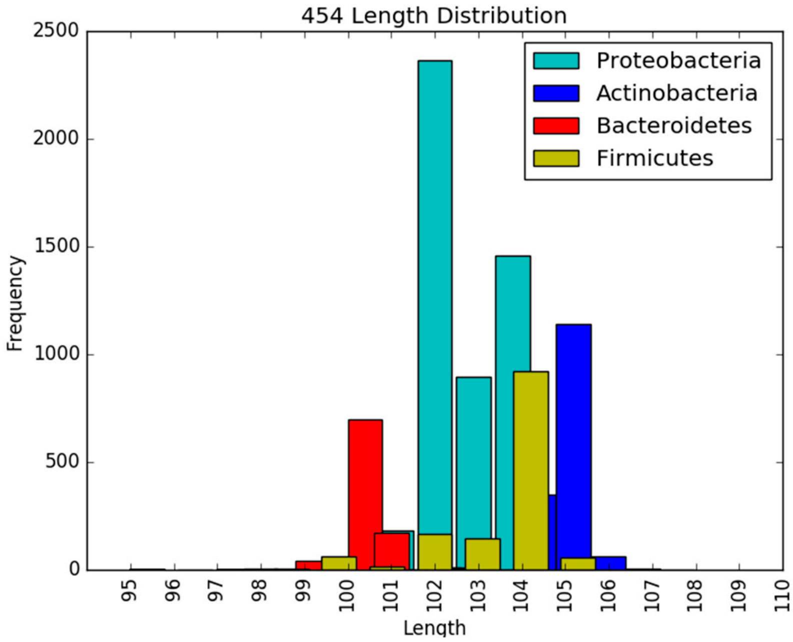

Figure S2. Length distribution for the 454 sample in Figure 6 (colors as in Figure 6B).

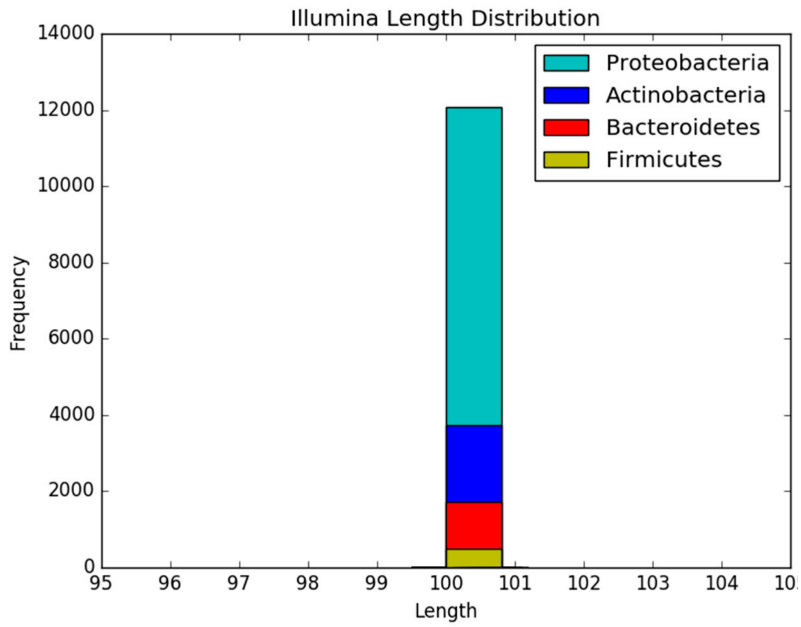

Figure S3. Length distribution for the Illumina sample in Figure 5 (colors as in Figure 5B).

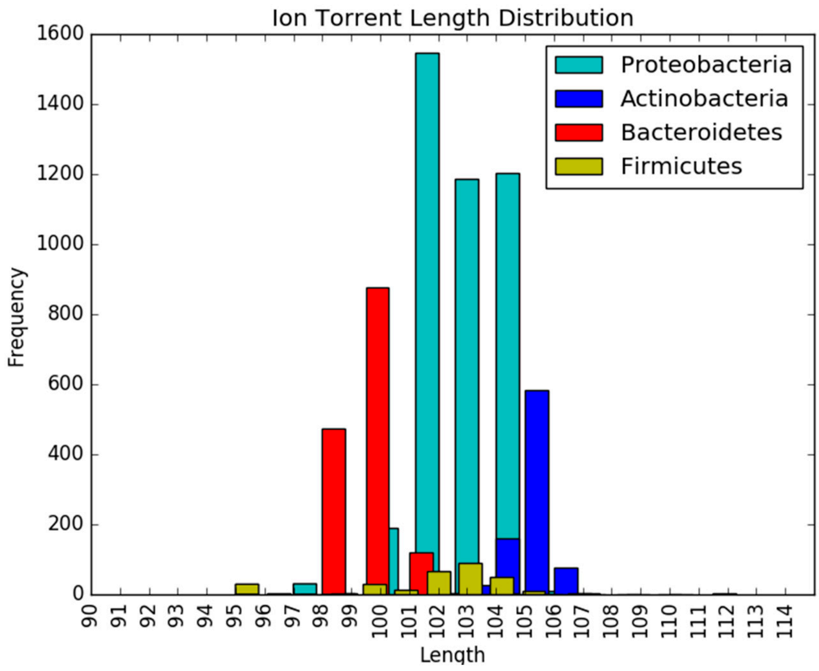

Figure S4. Length distribution for the Ion Torrent sample in Figures 5 and 6 (colors as in Figures 5B and 6B).

© 2017 by the authors. Submitted for possible open access publication under the terms and conditions of the Creative Commons Attribution (CC BY) license (http://creativecommons.org/licenses/by/4.0/).
